# ALDH1A1 overexpression is associated with the progression and prognosis in gastric cancer

**DOI:** 10.1186/1471-2407-14-705

**Published:** 2014-09-24

**Authors:** Xiao-shan Li, Qing Xu, Xiang-yang Fu, Wei-sheng Luo

**Affiliations:** Department of Gastroenterology, Affiliated Hospital of Guilin Medical University, Guilin, 541004 Guangxi Zhuang Autonomous Region China; Guilin Medical University, Guilin, 541004 Guangxi Zhuang Autonomous Region China; Department of Spleen and stomach diseases, The First Affiliated Hospital of Guangxi University of Chinese Medicine, Nanning, 530200 Guangxi Zhuang Autonomous Region China

## Abstract

**Background:**

Aldehyde dehydrogenase 1 family member A1 (ALDH1A1) is a cancer stem cell marker, and its expression correlates with prognosis in a number of malignancies. The aim of this study is to determine the relationship of ALDH1A1 expression with clinicopathological parameters and prognosis in gastric cancer.

**Methods:**

ALDH1A1 and matrix metallopeptidase 9 (MMP-9) was evaluated by immunohistochemistry in 216 gastric carcinoma samples. The association between expression of ALDH1A1 and MMP-9, clinicopathological parameters, and prognosis of gastric cancer was examined.

**Results:**

ALDH1A1 protein expression was significantly associated with depth invasion, lymph node metastasis and stage of disease (all *P* < 0.05). Both univariate and multivariate analyses revealed that ALDH1A1 was an independent prognostic factor for both overall survival (OS) and recurrence-free survival (RFS) (both *P* < 0.001). Furthermore, ALDH1A1 overexpression was associated with poor prognosis in patients subgroups stratified by tumor size, depth invasion and lymph node metastasis. Moreover, ALDH1A1 was significantly correlated with MMP-9 among 216 gastric cancer tissues (*P* < 0.001). Patients who had ALDH1A1 overexpression, in which tumor cells displayed high invasiveness, had poor OS and shorter RFS.

**Conclusion:**

ALDH1A1 plays an important role in tumor aggressiveness and prognosis, and may act as a promising target for prognostic prediction.

## Background

Gastric cancer is one of the leading causes of cancer-related death worldwide due to its frequency, poor prognosis and limited treatment options [1]. Complete resection of the tumor and adjacent lymph nodes is the only effective curative treatment [2]. Unfortunately, after a complete resection, the 5-year survival rate remains low [3]. Several studies have shown that various genetic and epigenetic alterations are involved in the course of carcinogenesis and progression of gastric cancer [4–6]. However, the molecular mechanism involved in the development of gastric cancer remains unclear.

Aldehyde dehydrogenases (ALDHs) are a group of proteins that share highly conserved sequences essential for function. Each subunit contains a catalytic domain, a cofactor binding domain, and a bridging domain. Each subtype’s catalytic pocket has a specificity for a particular substrate [7]. The human ALDH superfamily currently consists of 19 known putatively functional genes in 11 families and 4 subfamilies with distinct chromosomal locations [7–9]. The ALDH enzymes can be found in the cytosol, nucleus, mitochrondria, and endoplasmic reticulum. The function of ALDH is to modulate several cell functions, including proliferation, differentiation, and survival, as well as the cellular response to oxidative stress. It has been reported that the ALDH enzymes that are involved in normal stem cells as well as cancer stem cells include the ALDH1 family, ALDH2*2, ALDH3A1, ALDH4A1 and ALDH7A1 [7]. In particular, ALDH1 has been used as a marker to identify and isolate normal and cancer stem cells. It has been known that the ALDH1 subfamily comprises of ALDH1A1, ALDH1A2 and ALDH1A3.

ALDH1A1 is a cytosolic enzyme responsible for oxidizing a variety of intracellular aldehydes to carboxylic acids [10]. It also plays an important role in the detoxification of peroxidic aldehydes produced by ultraviolet light absorption, protecting the lens of the eye. In addition, it exhibits high activity for oxidation of aldophosphamide and has a role in the detoxification of some commonly used anticancer drugs [11]. Recently, it has been reported that ALDH1A1 has been related to adverse prognosis in several human malignancies, including breast cancer, lung cancer, ovarian cancer and esophageal cancer [12–15]. However, the role of ALDH1A1 on the prognosis of patients with gastric cancer remains unclear.

In the present study, we assessed the expression of ALDH1A1 in gastric cancer tissues by immunohistochemistry. Correlation of ALDH1A1 with clinicopathological parameters and survival of gastric cancer patients were then analyzed. In addition, it has been reported that MMP-9 plays an important role in gastric cancer recurrence and prognosis [16]. Therefore, we also investigated the relationship of ALDH1A1 and MMP-9 protein in gastric cancer.

## Methods

### Patients and specimens

The study was approved by the Institutional Review Board and Human Ethics Committee of Affiliated Hospital of Guilin Medical University. Written consent for using the samples for research purposes was obtained from all patients prior to surgery.

Gastric carcinoma tissues were obtained from gastrectomy specimens of 216 patients from the department of general surgery, the Affiliated Hospital of Guilin Medical University (Guilin, China). All the operations were performed between January 2005 and December 2008. The eligibility criteria of the current study were as follows: (1) a pathologic examination confirming the presence of gastric cancer and experienced radical surgery, (2) complete basic clinical data, (3) the absence of any prior treatment for cancer, and (4) no serious complications or other malignant disease. There were 140 males and 76 females (mean age, 57.0 years; range, 22–82 years). Relevant clinical pathologic features (Table 1) were all obtained from the patients’ files. Tumor stage was classified according to the 7th Union International Cancer Control (UICC) TNM staging system [17].

**Table 1:** **Clinicopathologic correlation of ALDH1A1 expression in 216 gastric cancer**

Characteristics	No. of patients	ALDH1A1 expression (%)		***P***-value
		Negative	Positive	
Gender				
Male	140	64 (45.7%)	76 (54.3%)	
Female	76	44 (57.9%)	32 (42.1%)	0.087
Age (years)				
≤ 60	136	74 (54.4%)	62 (45.6%)	
> 60	80	34 (42.5%)	46 (57.5%)	0.091
Size (cm)				
≤ 5.0	139	76 (54.7%)	63 (45.3%)	
> 5.0	77	32 (41.6%)	45 (58.4%)	0.065
Tumor site				
Upper	86	36 (41.9%)	50 (58.1%)	
Middle/Lower	130	72 (55.4%)	58 (44.6%)	0.052
Differentiation				
Well/Moderate	96	41 (42.7%)	55 (57.3%)	
Poor	120	67 (55.8%)	53 (44.2%)	0.055
Depth of invasion				
T1/T2	81	58 (71.6%)	23 (28.4%)	
T3/T4	135	50 (37.0%)	85 (63.0%)	< 0.001
Lymph node metastasis				
Negative	59	46 (78.0%)	13 (22.0%)	
Positive	157	62 (39.5%)	95 (60.5%)	< 0.001
Stages				
I/II	71	52 (73.2%)	19 (26.8%)	
III	145	56 (38.6%)	89 (61.4%)	< 0.001

### Immunohistochemistry staining

A total of 216 gastric carcinoma samples were used in the immunohistochemistry (IHC) analysis. According to protocol [18] for IHC on paraffin-embedded tissue sections, paraffin-embedded blocks were sectioned at about 4 μm thickness. Slides were baked at 60°C for 2 h, deparaffinized with xylene and rehydrated using an alcohol gradient (100% alcohol, 95% alcohol, 80% alcohol, and 70% alcohol). The tissue slides were then treated with 3% hydrogen peroxide in methanol for 30 min to quench endogenous peroxidase activity, and the antigens were retrieved in 0.01 M sodium citrate buffer (pH 6.0) using a microwave oven. After 30 min of preincubation in 10% normal goat serum to prevent nonspecific staining, the samples were incubated overnight using a primary antibody, either anti-ALDH1A1 (Abcam, #ab52492, UK, dilution 1:200) or anti-MMP-9 (Abcam, #ab38898, UK, dilution 1:200), in a humidified container at 4°C. The tissue slides were treated with a non-biotin horseradish-peroxidase detection system according to the manufacturer’s instructions (Gene Tech). The IHC results were evaluated by two independent investigators blinded to the patients’ identity and clinical status. In discrepant cases, a pathologist reviewed the cases, and a consensus was reached.

**Figure 1:**
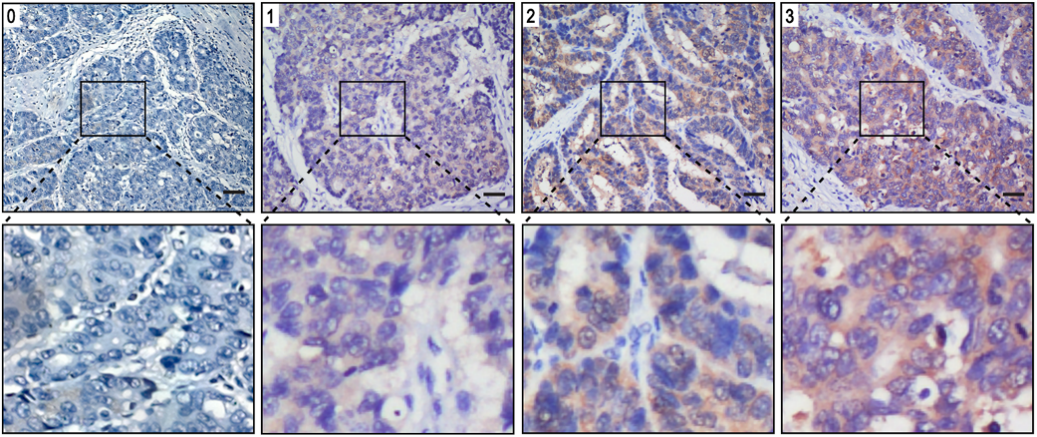
**Gastric cancer tissue illustrating the range of intensities of ALDH1A1 immunostaining from 0 to 3.** The lower panels represent magnified pictures of boxed area in the corresponding upper panels. The scale bar represents 50 μm.

ALDH1A1 and MMP-9 staining intensities were rated on a scale of 0–3 according to the percentage of positive tumor (0, < 5% positive cells; 1, 5-10%; 2, 11-50%; or 3, > 50%). The expression is very low for 0, low for 1, moderate for 2 and high for 3 (Figure 1). ALDH1A1 and MMP-9 expression were classified as negative for scores ≤ 1 and positive for scores ≥ 2.

### Follow-up

The follow-up duration was defined as the interval between the date of operation and the date of death or last follow-up. The study was censored on 30 September 2013. The median follow-up period was 27.0 months (range, 4–82 months) in 216 patients. All the patients were followed up every 1–3 months in the first year and every 3–6 months thereafter. Recurrence were confirmed by tumor markers levels including CEA, AFP, CA199, CA125 and CA724, B-type ultrasonic inspection every 3 moths, and computed tomography (CT) or magnetic resonance imaging (MRI) every 6 months after gastrectomy. The main causes of death were gastric cancer recurrence. Overall survival (OS) was calculated from the date of surgery to the date of death or last follow-up. Recurrence-free survival (RFS) was defined as from the date of surgery until the date of relapse or from the period of resection to the date of the last observation taken.

### Statistical analysis

All statistical analyses were performed using the SPSS software (version 16.0; Chicago, IL, USA). Interdependence between ALDH1A1 status and clinical data was calculated using the chi-square test, and displayed in cross-tables. Correlation of ALDH1A1 with MMP-9 was calculated by Pearson χ2 test. Survival curves were plotted using the Kaplan-Meier method and analyzed using the log-rank test. All reported *P* values were two-sided and *P* < 0.05 was considered statistically significant.

## Results

### The association of ALDH1A1 with clinicopathological variables

To elucidate the biological significance of ALDH1A1 in gastric cancer, we examined the immunohistochemical expression of ALDH1A1 in gastric cancer tissues (Figure 1). ALDH1A1 staining mainly located in cytoplasm of tumor cells. The positive rate of ALDH1A1 was 50.0% (108/216) in gastric cancer samples.

According to the results of immunohistochemistry, we correlated ALDH1A1 status in 216 gastric cancer specimens with clinicopathologic parameters (Table 1). Our analyses showed that the level of ALDH1A1 in gastric cancer was significantly correlated with depth of invasion (*P* < 0.001), lymph node metastasis (*P* < 0.001), and stage of disease (*P* < 0.001), but was not associated with gender, age, tumor size, tumor site and grade of differentiation (*P* > 0.05) (Table 1). Notably, the correlation of ALDH1A1 with prominent serosal invasion and lymph node metastasis positivity suggested a potential role of ALDH1A1 in increased invasion and metastasis of gastric cancer.

### Effect of tumor ALDH1A1 protein level on prognosis

To further determine the effect of ALDH1A1 overexpression on the OS and RFS, we first performed univariate analysis of traditional clinicopathologic variables for prognosis. The results of the univariate analysis are shown in Table 2. Overexpression of ALDH1A1 (*P* < 0.001), larger tumor size (*P* < 0.001), prominent serosal invasion (*P* < 0.001) and lymph node metastasis (*P* < 0.001) were significantly associated with the poor OS rate of gastric cancer patients. In addition, Kaplan-Meier analysis demonstrated that ALDH1A1 overexpression (*P* < 0.001), larger tumor size (*P* = 0.001), tumor site (*P* = 0.047), prominent serosal invasion (*P* < 0.001) and lymph node metastasis (*P* < 0.001) were negative prognostic factors for RFS in gastric cancer patients (Table 2). Furthermore, to evaluate the independent impact of ALDH1A1 overexpression on OS and RFS, a multivariate Cox regression model adjusted for tumor size, tumor site, depth of invasion, lymph node metastasis and ALDH1A1 expression was performed. Our results showed that ALDH1A1 expression was a poor independent prognostic factor for OS in gastric cancer patients (hazard ratio, 2.037; 95% CI, 1.407 - 2.950). In addition, positive ALDH1A1 expression patients were almost 2.0 times more likely to suffer from relapse than those with negative ALDH1A1 expression (hazard ratio, 1.945; 95% CI, 1.346 - 2.812). Tumor size, depth of invasion and lymph node metastasis all had independent prognostic value in the multivariate analysis (Table 3).

**Table 2:** **Predictive variables for overall survival and recurrence-free survival of 216 patients with gastric cancer**

Variables	No. of patients	OS rate (%)		***P***-value	RFS rate (%)		***P***-value
		3 y	5 y		3 y	5 y	
Gender							
Male	140	46.9	39.6		41.5	35.4	
Female	76	54.0	43.1	0.308	44.4	41.2	0.381
Age (years)							
≤ 60	136	49.8	43.9		45.3	42.2	
> 60	80	48.9	36.1	0.338	38.1	30.6	0.391
Size (cm)							
≤ 5.0	139	55.6	47.4		50.4	45.5	
> 5.0	77	38.1	28.8	0.001	28.3	23.3	< 0.001
Tumor site							
Upper	86	45.9	33.8		34.6	28.6	
Middle/Lower	130	51.8	45.7	0.074	47.8	43.7	0.047
Differentiation							
Well/Moderate	96	47.8	39.7		41.2	34.6	
Poor	120	50.7	41.6	0.673	43.6	40.1	0.822
Depth of invasion							
T1/T2	81	71.4	66.5		70.2	65.3	
T3/T4	135	36.2	25.5	< 0.001	25.6	20.8	< 0.001
Lymph node metastasis							
Negative	59	83.5	79.2		82.0	77.5	
Positive	157	36.5	26.6	< 0.001	27.5	22.6	< 0.001
ALDH1A1 protein expression							
Negative	108	69.3	57.1		60.5	54.8	
Positive	108	28.7	23.9	< 0.001	23.7	19.2	< 0.001

**Table 3:** **Cox multivariate analysis of contributory factors to prognosis among 216 gastric cancer patients after gastrectomy**

Variables	β	SE	Hazard ratio (95% CI)	***P***-value ^a^
OS				
Tumor size	0.410	0.177	1.507 (1.065 ~ 2.133)	0.021
Depth of invasion	0.576	0.255	1.779 (1.079 ~ 2.931)	0.024
Lymph node metastasis	1.018	0.341	2.767 (1.417 ~ 5.403)	0.003
ALDH1A1 protein expression	0.712	0.189	2.037 (1.407 ~ 2.950)	< 0.001
RFS				
Tumor size	0.411	0.178	1.508 (1.064 ~ 2.138)	0.021
Tumor site	0.043	0.180	1.044 (0.733 ~ 1.486)	0.813
Depth of invasion	0.649	0.256	1.913 (1.158 ~ 3.162)	0.011
Lymph node metastasis	1.063	0.338	2.894 (1.491 ~ 5.619)	0.002
ALDH1A1 protein expression	0.665	0.188	1.945 (1.346 ~ 2.812)	< 0.001

*Abbreviations*: *ALDH1A1* aldehyde dehydrogenase 1 family member A1, *CI* confidence interval.

^a^Cox proportional hazards regression model.

**Figure 2:**
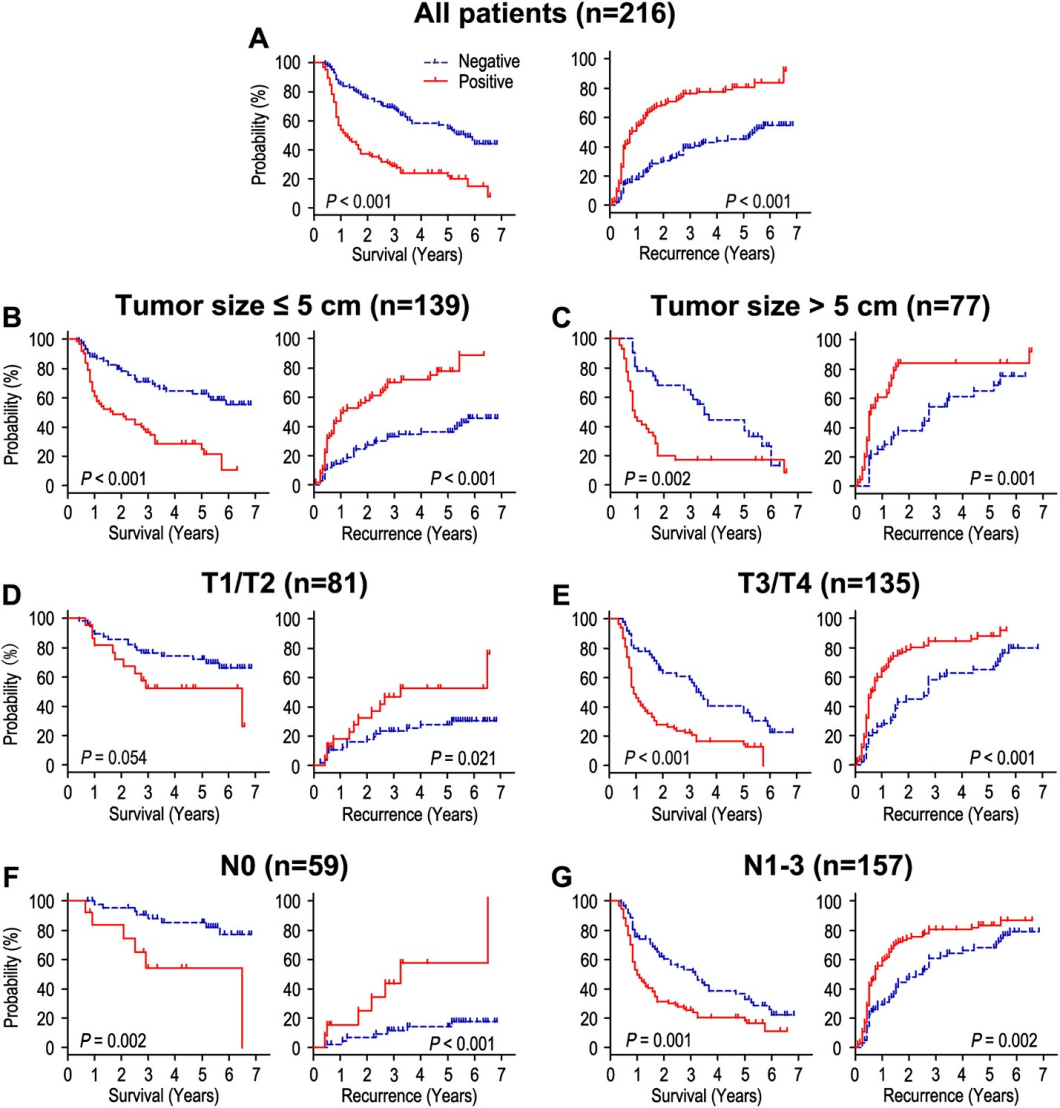
**Overall survival and recurrence-free survival are shown for patients with gastric cancer.** All patients were stratified according to tumor size, depth of invasion and lymph node metastasis. Kaplan-Meier survival estimates and log-rank tests were used to analyze the prognostic significance of ALDH1A1 expression in all patients **(A)** and each subgroup **(B-G)**.

Survival analysis showed that OS and RFS were significant different among 216 patients according to the expression of ALDH1A1 (*P* < 0.001, *P* < 0.001) (Figure 2A). The postoperative median OS and RFS were 27.0 months and 19.0 months, respectively. The postoperative median OS times in ALDH1A1-positive (n = 108) and ALDH1A1-negative (n = 108) gastric cancer patients subgroup were 12.0 months and 42.0 months, and the median of the RFS times were 9.0 months and 39.0 months. In addition, the OS and RFS rates at 5 years were 23.9% and 19.2% for ALDH1A1-positive patients compared with 57.1% and 54.8% for ALDH1A1-negative patients, respectively (both *P* < 0.001; Table 2).

To further evaluate the prognostic value of ALDH1A1 in different subgroups, patients were stratified according to tumor size (Figure 2B,C), depth of invasion (Figure 2D,E) and lymph node metastasis (Figure 2F,G). The expression of ALDH1A1 maintained its prognostic value in predicting shorter OS and RFS in all of these subgroups for except OS in T1/T2 subgroup (*P* = 0.054). Therefore, it appears that ALDH1A1 may serve as a powerful prognostic factor for patients with gastric cancer in different risk groups.

### ALDH1A1 overexpression predict poor prognosis independent of tumor invasiveness

To better understand the clinical significance of ALDH1A1 on aggressiveness in gastric cancer, we investigated the relationship of ALDH1A1 and MMP-9 protein expression in gastric cancer.

**Figure 3:**
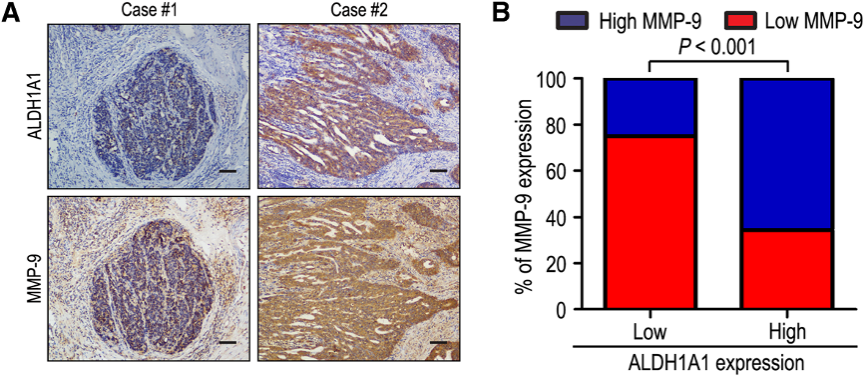
**ALDH1A1 and MMP-9 levels correlated in 216 gastric cancer tissues. (A, B)** IHC staining for ALDH1A1 and MMP-9 was performed in tumors from 216 gastric cancer patients. Representative examples of ALDH1A1 and MMP-9 staining in serial sections from the same tumor samples are shown in **(A)**, and percentages of samples displaying low or high ALDH1A1 expression relative to MMP-9 level is shown in **(B)**. The scale bar represents 200 μm.

The positive rates of ALDH1A1 were 63.0% and 60.5% in the more prominent serosal invasion group (T3/T4) and more frequent lymph node involvement group (N1-3), while there were only 28.4% and 22.0% in T1/T2 and N0 (*P* < 0.001 and *P* < 0.001, respectively) (Table 1). In addition, ALDH1A1 was significantly correlated with MMP-9 in 216 gastric carcinoma specimens. Of 108 patients with low ALDH1A1 expression, 81 patients (75.0%) had low MMP-9 expression, while 71 of 108 patients (65.7%) with high ALDH1A1 expression also had high MMP-9 expression (*P* < 0.001) (Figure 3).

**Figure 4:**
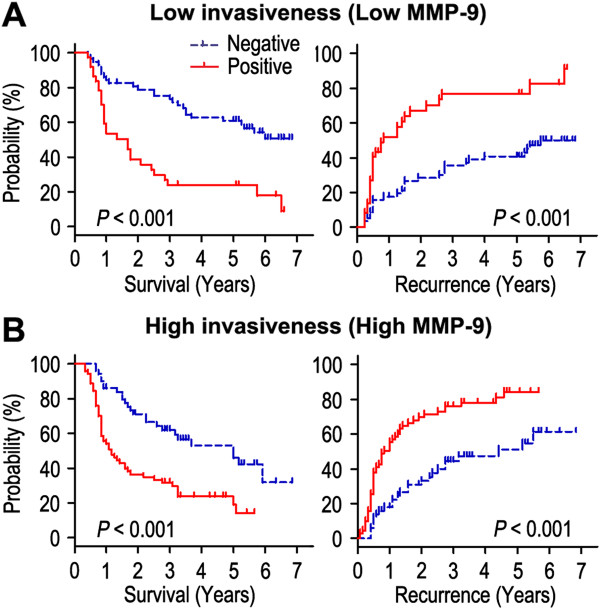
**Overall survival and recurrence-free survival are shown for patients with low tumor invasiveness (A) and high tumor invasiveness (B).** Kaplan-Meier survival estimates and log-rank tests were used to analyze the association between ALDH1A1 expression and overall survival or recurrence-free survival in patients with low invasiveness (low MMP-9; n = 118) or high invasiveness (high MMP-9; n = 98).

We further explored the influence of tumor invasiveness on the prognostic value of ALDH1A1 expression in gastric cancer by using MMP-9 as an indicator for the invasive potential of individual tumor cells. All the patients were stratified into either a low invasiveness subgroup (low MMP-9 expression; n = 118) or a high invasiveness subgroup (high MMP-9 expression; n = 98) according to the MMP-9 expression index. Kaplan-Meier survival curves were then plotted to investigate the association between ALDH1A1 status and survival (Figure 4). In the low invasiveness subgroup, ALDH1A1 overexpression was associated with shorter OS (*P* < 0.001) and RFS (*P* < 0.001) compared with the OS and RFS in patients with low ALDH1A1 expression (Figure 4A). In the high tumor invasiveness subgroup (Figure 4B), patients with ALDH1A1 overexpression were prone to death (*P* < 0.001) and relapse (*P* < 0.001). Furthermore, the 5-year survival rate was significantly lower in the low invasiveness subgroup with ALDH1A1 overexpression (23.8%) than that in the high invasiveness subgroup with low ALDH1A1 expression (53.0%; *P* = 0.002; data not shown). Therefore, the expression of ALDH1A1 appears to be a strong postoperative prognostic parameter for patients with gastric cancer independent of tumor invasiveness.

## Discussion

In the present study, the expression of ALDH1A1 was investigated in 216 gastric carcinoma tissues by immunohistochemistry. We found that ALDH1A1 was significantly associated with depth invasion, lymph node metastasis and stage of disease. In addition, the Kaplan-Meier survival analysis revealed that the survival times (OS and RFS) of gastric cancer patients with high ALDH1A1 expression were significantly shorter than those with low ALDH1A1 expression. The prognostic value of ALDH1A1 in different subgroups according to tumor size, depth of invasion and lymph node metastasis was also estimated, which appears that ALDH1A1 may serve as a powerful prognostic factor for patients with gastric cancer in different risk groups. Furthermore, the multivariate Cox model analysis indicated that ALDH1A1 status was an independent factor for both prognosis indexes (OS and RFS) in gastric cancer. This finding suggests that ALDH1A1 plays an important role in tumor prognosis, concludes ALDH1A1 could be a potential prognostic factor of gastric cancer. Our results were consistent with previously reported results. In several investigations, it has been shown that the abnormal expression of ALDH1A1 in cancer cells is associated with tumor progression. Wakamatsu et al. [19] revealed that ALDH1 was overexpression and had positively correlated with depth invasion and TNM stage in gastric cancer, moreover, ALDH1 positivity was significantly higher in diffuse-type lymph node metastasis than that in the primary tumor. Charafe-Jauffret et al. [20] reported that the ALDH1A1-positive breast cancer cells were able to promote tumor invasion *in vitro* and tumor metastasis in mouse xenografts, moreover, expression of ALDH1A1 was an independent predictive factor for early metastasis and decreased survival in inflammatory breast cancer. Jiang et al. [14] showed that the ALDH1A1-positive lung cancer cells could generate tumors *in vivo*, furthermore, the expression of ALDH1A1 was positively correlated with the stage and grade of lung tumors and related to a poor prognosis for the patients with early-stage lung cancer, which suggested that ALDH1A1 could be a potential prognostic factor and therapeutic target for treatment of the patients with lung cancer. However, Dimou et al. [21] found the contradictory results that the ALDH1A1-negative expression of lung cancer patients had shorter survival compared with those with ALDH1A1-positive expression, which indicated that ALDH1A1 overexpression was associated with favorable outcome.

It has been known that degradation of extracellular matrix (ECM) was a signal for the beginning of invasion and metastasis, and MMPs are important molecules involved in ECM degradation during invasion and metastasis [22]. Chu et al. [16] reported that cancer MMP-9 was significantly correlated with depth of invasion and lymph node metastasis and MMP-9-positive gastric cancer patients had worse outcomes than those with MMP-9-negative tumors. Zhao et al. [23] found that MMP-9 targeted RNA interference was able to successfully suppress MMP-9 expression and inhibit cell growth and invasion of SGC7901 gastric cancer in vitro and in vivo. Our results demonstrated that the expression of ALDH1A1 and MMP-9 was correlated with each other, indicating higher invasive and metastasizing activity in ALDH1A1 overexpression cancer cells. In addition, ALDH1A1 was highly expressed in depth of invasion, especially in T3 and T4 carcinomas, which was consistent with previously reported results [19]. As far as lymph node status was concerned, the patients with lymph node metastasis tend to show elevated ALDH1A1 expression. Collectively, ALDH1A1 status in gastric cancer promoting tumor aggressiveness suggests that ALDH1A1 could be a feasible target in cancer therapy.

## Conclusions

In this study, we demonstrated that ALDH1A1 may play an important role in tumor invasion, metastasis and prognosis, and could work as a promising target for prognostic prediction in gastric cancer. Determination of ALDH1A1 expression may help to identify high-risk gastric cancer patients and thus aid the selection of appropriate therapies. Further investigation is necessary to clarify the role of ALDH1A1 in the development of gastric cancer.
